# Supplementary material for: Leucine-Rich Alpha-2-Glycoprotein: A Novel Predictor of Diastolic Dysfunction
Source: Biomedicines. 2023 Mar 20;11(3):944. doi: 10.3390/biomedicines11030944 (PMC10045934; doi:10.3390/biomedicines11030944)
Supplement: Supplementary file 1 [file biomedicines-11-00944-s001.zip › biomedicines-2216627-supplementary.pdf]

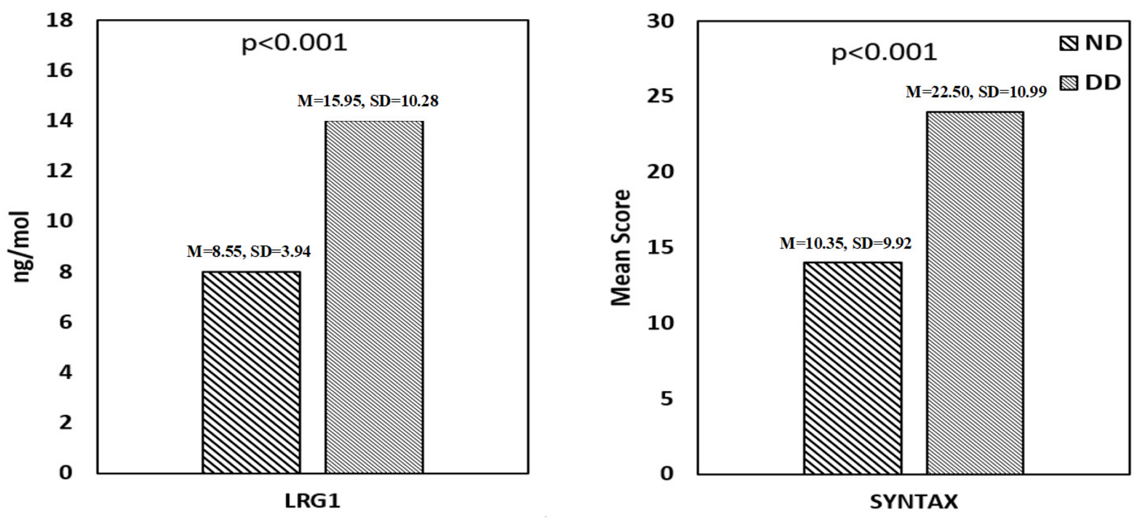

Supplementary Figure S1. Pairwise comparison of median of serum LRG1 levels and SYNTAX between No-DD and DD groups. Pairwise groups comparison was performed with Mann Whitney U Test. P<0.05* was statistically significant. SYNTAX = Synergy Between Percutaneous Coronary Intervention with Taxus and Cardiac Surgery. No-DD = without diastolic dysfunction, DD = with diastolic dysfunction, M = Mean, SD = standard deviation.
